# Adverse obstetric outcome and its associated factors in public hospitals of North Ethiopia: does parity make a difference?

**DOI:** 10.1186/s12884-022-05021-2

**Published:** 2022-09-08

**Authors:** Mesfin Tadese, Saba Desta Tessema, Birhan Tsegaw Taye, Getaneh Baye Mulu

**Affiliations:** 1 Department of Midwifery, School of Nursing and Midwifery, Asrat Woldeyes Health Science Campus, Debre Berhan University, Debre Berhan, Ethiopia; 2 Department of Nursing, School of Nursing and Midwifery, Asrat Woldeyes Health Science Campus, Debre Berhan University, Debre Berhan, Ethiopia; 3 Department of Epidemiology, University Medical Centre of Groningen, University of Groningen, Groningen, the Netherlands

**Keywords:** Adverse obstetric outcome, Grand multipara, Low multipara, Comparative study

## Abstract

**Background:**

Direct obstetric causes account for nearly 75% of all maternal deaths. Controversy prevails in the effect of grand multiparity on adverse obstetric outcomes. This study thus aimed to determine and compare the obstetric outcomes in low multiparous (LM) and grand multiparous (GM) women in Public Hospitals of North Ethiopia.

**Method:**

An institution-based comparative cross-sectional study was done among 540 (180 GM and 360 LM) participants from January 1 to March 30, 2021. The data was collected through face-to-face interviews and a review of clinical records and birth registries. Epi-Data version 4.6 was used for data entry and analysis was performed using SPSS version 25.0 statistical software. A *p*-value of ≤ 0.05 (2-tailed) was used to consider the significance of statistical tests.

**Result:**

The prevalence of adverse obstetric outcomes was 32.6% (95% CI: 28.7–36.5). Antepartum hemorrhage, anemia, and postpartum hemorrhage were higher in grand multiparous women. Whereas, prolonged labor, induction/augmentation, prelabor rupture of membrane, episiotomy, and post-term pregnancy was higher in low multiparous women. Income (AOR (CI) = 3.15 (1.30–7.63), alcohol consumption (AOR (CI) = 3.15 (1.49–6.64), preterm delivery (AOR (CI) = 9.24 (2.28–27.3), cesarean delivery (AOR (CI) = 13.6 (6.18–30.1), and low birth weight (AOR (CI) = 3.46 (1.33–9.03) significant predictors of adverse obstetric outcomes. However, parity did not show a statistically significant difference in obstetric outcomes.

**Conclusion:**

In the study area, obstetric complications were high compared to a systematic review and meta-analysis study done in the country (26.88%). Socio-economic status, alcohol consumption, gestational age at delivery, mode of delivery, and birth weight were significant associates of the obstetric outcome. There was no statistically significant difference in obstetric outcomes between GM and LM women. Socio-economic development, avoiding alcohol consumption, early identification and treatment of complications, and adequate nutrition and weight gain during pregnancy are needed regardless of parity.

## Introduction

Globally, in 2017, an estimated 295,000 women died during pregnancy, delivery, and the postpartum period. Most (94%) of all deaths occurred in low and lower-middle-income countries such as Ethiopia. Sub-Saharan Africa accounted for nearly 66% of the global maternal deaths, while Southern Asia accounted for about 20% [1]. However, maternal deaths only tell part of the story. For every single woman who dies of pregnancy-related complications, between 20 and 30 more suffer short- and long-term disabilities, such as infections, hemorrhage, obstetric fistula, uterine rupture, or pelvic inflammatory disease [2]. In Ethiopia, more than 500,000 women and girls suffer from disabilities as a result of complications during pregnancy and childbirth each year [2]. According to a WHO report, pregnancy complications account for nearly 75% of all maternal deaths [1]. The top causes of maternal deaths in In Ethiopia are hemorrhage (29.9%), obstructed labor (22.3%), pregnancy-induced hypertension (16.9%), puerperal sepsis (14.68%), and unsafe abortion (8.6%) [3].

A prospective cohort study in Bangladesh reported a 25% magnitude of obstetric complications [4]. In Uganda, one-third of women reported an adverse pregnancy outcome [5]. Similarly, a comparative study in Southern Ethiopia determined the prevalence of adverse obstetric outcomes at 39%. The study further reported a higher prevalence of hypertensive disorders of pregnancy, antepartum hemorrhage (APH), and premature rupture of membrane (PROM) among grand multiparous women, while the higher risk of obstructed labor and cesarean delivery among low multiparous women [6]. Extreme ages and a history of stillbirth/miscarriage significantly increased the risks of obstetric complications [4]. History of medical illnesses, previous cesarean delivery, and high birth weight were also significant factors of adverse obstetric outcomes [6].

Various studies have investigated the impact of grand multiparity on adverse obstetric and perinatal outcomes, and mixed findings were reported. Some studies showed an increased incidence of obstetric complications among grand multiparas, however, other studies explored a comparable risk of complications. A study in Saudi Arabia found that grand multiparous women have a comparable risk of maternal and neonatal complications compared to other parity groups [7]. Similarly, a comparative study in Southern Ethiopia found that parity did not show a statistically significant difference in obstetric outcomes [6]. However, grand multiparity was found to be a risk factor for PROM, stillbirth, and preterm delivery in Tanzania [8]. It was also associated with adverse maternal outcomes like cesarean delivery, fetal macrosomia, diabetes mellitus, and pregnancy-induced hypertension [9].

Sustainable development goal 3.1 sets a target for all global nations to decrease the maternal mortality ratio to less than 70 by 2030 [10]. Furthermore, the Global Strategy for Women’s, Children’s, and Adolescents’ Health planned to design programs aimed at maternal and child health globally [11]. Although older literature showed the effect of parity on maternal and perinatal outcomes, recent reports fail to support these findings. Hence, this study planned to determine and compare adverse obstetric outcomes in grand multiparous (GM) and low multiparous (LM) women and identify its associated factors in public hospitals of North Ethiopia.

## Methods

### Study design, setting, and period

An institution-based comparative cross-sectional study was done in North Shewa Zone public hospitals from January 1 to March 30, 2021. North Shewa is one of the 10 zones in the Amhara region of Ethiopia and is 130 km far from Addis Ababa, the capital of Ethiopia. Based on the 2007 national census, it has a total population of 1,837,490; 928,694 men and 908,796 women [12]. There are 12 Hospitals (2 private, 9 public, and 1 comprehensive specialized hospital). There are about 303 midwives, 766 Nurses, 120 Laboratory professionals, 130 Pharmacists, 150 General Practitioners, 15 Specialists, 25 integrated emergency surgical officers (IESO), and 54 Anesthesia professionals working in the zone.

### Study population and inclusion and exclusion criteria

All multiparous women who gave birth in the study area were the source population. Randomly selected multiparous women in labor wards during the study period were the study population. Multiparous women with a single fetus/neonate at a gestational age of 28 weeks or above were included. Multiparous women with twin gestation/delivery, with known medical conditions like diabetes, HIV, and hypertension, referred from other health institutions, home delivery, and those who were unable to communicate or seriously ill were excluded from the study.

### Sample size calculation

The sample size was computed using Open-Epi version 3.03 statistical software. The following assumptions were made: the power of the study (1-β) to be 80%, 95% confidence interval (CI), the estimated ratio of unexposed (LM)-to exposed (GM) ratio is 2:1, and the percent of outcome among non-exposed group and odds ratio of previous studies were used as indicated in Table 1 below. Adding a 10% non-response rate, the largest sample size was 548 (183 GM and 365 LM).

**Table 1:** Sample size calculation for adverse obstetric outcomes in low multiparous and grand multiparous women who give birth in North Shewa Zone Hospitals, 2021

Variables	% of outcome in unexposed group	Adjusted odds ratio	Sample size GM/LM [Total]	Reference
Anemia	16.8	3.5	44/88 [132]	[13]
Home delivery	20.66	1.87	166/332 [**498**]	[14]
Preterm delivery	5.8	5.3	47/93 [140]	[15]
Cesarean section	37.9	2.7	55/110 [165]	[9]

### Sampling procedure

**Fig. 1:**
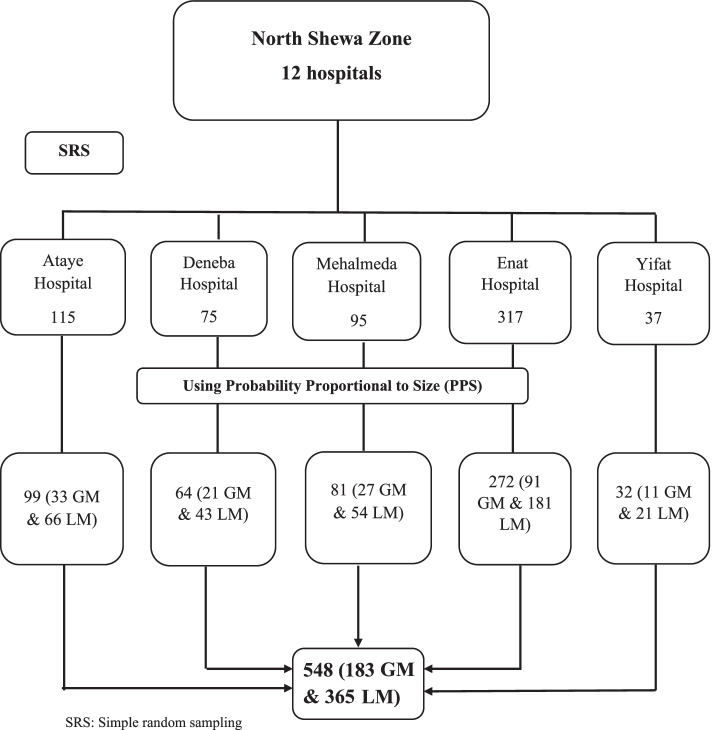
Sampling procedure for the assessment of adverse obstetric outcomes in low multiparous and grand multiparous women who give birth in North Shewa Zone Hospitals, 2021

Simple random sampling was applied to select 5 hospitals. The average number of deliveries in these hospitals was estimated to be 639 per month. The sample size was proportionally allocated to each hospital based on their respective number of deliveries (Fig. 1). For each grand multiparous woman, two low multiparous women from the same hospital were selected consecutively as they were present.

### Data collection tool and quality control

A structured and pre-tested questionnaire was used to obtain information from demographic characteristics, antenatal and obstetric history, and obstetric complications. The data was collected through face-to-face interviews and a review of clinical records and birth registries. A standard questionnaire was first prepared in English, then translated to the local language Amharic and back to English by an independent translator to keep the consistency of the instrument. The questionnaire was adapted from published articles [6, 9, 15] and modified according to the local context.

Data quality was assured during collection, coding, entry, and analysis. Three diploma and two BSc midwives conducted interviews on daily basis using a standardized questionnaire for all women who deliver at the hospital within one to two hours of birth or immediately after recovery from a cesarean/complicated delivery. A training was given to the data collectors and supervisors regarding the objective, methods, tool, and data collection procedure to avoid any confusion and have a common understanding of the study. A pretest was done on 27 mothers (5% of the samples; 9 GM and 18 LM) in Arerti primary hospital and necessary amendments were considered following the result. The supervisors and principal investigators follow the daily activities of data collectors and checked the completeness, consistency, and clarity of the data.

### Measurements

Low multiparity: a woman having two to four live births or stillbirths after 28 weeks of gestation [6, 15]. Grand multiparity: woman with five or more live births or stillbirths after 28 weeks of gestation [6, 15].

Obstetric outcomes: at least one obstetric complication before, during, and after delivery were considered, i.e., APH, PPH, anemia, PROM, preterm labor, cesarean section, uterine rupture, sepsis, pregnancy-induced hypertension, oligohydramnios, prolonged pregnancy, obstructed labor, induction/augmentation, and maternal death [6, 9].

### Data processing and analysis

After data was collected and extracted from records, it was cleaned, coded, and checked for consistency and verification of missing values. Data was entered into Epi-Data version 4.6 and analysis was performed using SPSS version 25.0 statistical software. Descriptive statistics were summarized using frequency tables, percentages, and figures. Cross-tabulation and bivariable and multivariable analysis of variables were computed. Independent variables with a *p*-value of less than 0.25 in the bivariable analysis were exported into the multivariable logistic regression analysis. Multivariable analysis was used to control the possible confounders and identify important predictors of adverse obstetric outcomes. The adjusted odds ratio (AOR) with a 95% confidence interval was used to measure the strength of association. A *p*-value of ≤ 0.05 (2-tailed) was used to consider the significance of statistical tests. The model fitness was checked using Hosmer–Lemeshow goodness-of-fit (*p* = 0.688).

## Result

### Demographic characteristics

A total of 540 (180 LM and 360 GM) women were included in this analysis with a response rate of 98.9%. About 306 (85%) of LM and 70 (38.9%) of GM women were within the age range of 21 – 34 years (*p* = 0.000). Three-fourths, 266 (74%) of LM women reside in urban areas, while half 94 (52.2%) of GM women were rural residents (*p* = 0.000). Besides, 158 (44%) of LM and 42 (23.3%) of GM women had attended higher education (*p* = 0.000), and 58 (16.1%) of LM and 62 (34.4%) of GM women married before 18 years (*p* = 0.000). Further, 60 (16.7%) of LM and 32 (17.8%) of GM women have changed partners (Table 2).

**Table 2:** Distribution of socio-demographic data by parity among women who gave birth in North Shewa Zone Hospitals, 2021

**Variables**	**Category**	**Parity, n (%)**		
		**LM (360)**	**GM (180)**	***p*-value**
**Age of the mother**	20 – 34 years < 20 years ≥ 35 years	306 (85.0) 8 (2.2) 46 (12.8)	70 (38.9) 4 (2.2) 106 (58.9)	0.000*
**Residence**	Rural Urban	94 (26.1) 266 (73.9)	94 (52.2) 86 (47.8)	0.000
**Religion**	Christian Muslim	304 (84.4) 56 (15.6)	120 (66.7) 60 (33.3)	0.000
**Mother’s education**	No formal education Primary Secondary Higher education	66 (18.3) 76 (21.1) 60 (16.7) 158 (43.9)	106 (58.9) 14 (7.8) 18 (10.0) 42 (23.3)	0.000
**Mother’s Occupation**	Housewife Gov’t employee Self-employed	164 (45.6) 112 (31.1) 84 (23.3)	108 (60.0) 26 (14.4) 46 (25.6)	0.000
**Marital status**	Married Others^a^	354 (98.3) 6 (1.7)	170 (94.4) 10 (5.6)	0.012
**Husband education**	No formal education Primary Secondary Higher education	74 (20.9) 68 (19.2) 26 (7.3) 186 (52.5)	86 (50.6) 14 (8.2) 10 (5.9) 60 (35.3)	0.000
**Husband occupation**	Farmer Gov’t employee Self-employed	116 (32.8) 130 (36.7) 108 (30.5)	98 (57.6) 44 (25.9) 28 (16.5)	0.000
**Age at marriage**	< 18 years ≥ 18 years	58 (16.1) 302 (83.9)	62 (34.4) 118 (65.6)	0.000
**Changing partner**	Yes No	60 (16.7) 300 (83.3)	32 (17.8) 148 (82.2)	0.746
**Income (ETB)**	Lower tertile Middle tertile Higher tertile	134 (37.2) 118 (32.8) 108 (30.0)	88 (48.9) 44 (24.4) 48 (26.7)	0.027

*ETB* Ethiopian Birr, *GM* Grand multiparous, *LM* Low multiparous

^a^ Single, divorced, and widowed

^* ^Fisher’s exact test

### Obstetric profile

The mean (± SD) gestational age was 39.17 ± 2.27 and 39.02 ± 2.09 weeks for low multiparous and grand multiparous women, respectively. About 342 (95.0%) of LM and 168 (93.3%) of GM women spaced a child for a minimum of two years. The majority of LM 348 (96.7%) and GM 156 (86.7%) women had antenatal care (ANC) follow-up. Abortion, neonatal mortality, and cesarean sections were the most frequently encountered obstetric complications previously. Additionally, 18 (5%) of LM mothers and 38 (21%) of GM mothers had previously given birth at home (Table 3).

**Table 3:** Obstetric characteristics of LM and GM women who gave birth in North Shewa Zone Hospitals, 2021

**Variables**	**Category**	**Parity, n (%)**		
		**LM (360)**	**GM (180)**	***p*-value**
**Interpregnancy interval**	< 24 months ≥ 24 months	18 (5.0) 342 (95.0)	12 (6.7) 168 (93.3)	0.425
**ANC visit**	Yes No	348 (96.7) 12 (3.3)	156 (86.7) 24 (13.3)	0.000
**GA at first ANC visit**	≤ 16 weeks > 16 weeks	218 (62.6) 130 (37.4)	60 (38.5) 96 (61.5)	0.000
**Number of ANC visits**	1 – 3 ≥ 4	118 (33.9) 230 (66.1)	68 (43.6) 88 (56.4)	0.037
**Past obstetric complications**	Yes No	136 (37.8) 224 (62.2)	102 (56.7) 78 (43.3)	0.000
**Types of complications**	Abortion Stillbirth/IUFD Neonatal mortality Preterm delivery Instrumental delivery Cesarean section Congenital anomaly	60 (16.7) 6 (1.7) 8 (2.2) 12 (3.3) 32 (8.9) 24 (6.7) 2 (0.6)	66 (36.7) 30 (16.7) 28 (15.6) 0 (0.0) 10 (5.6) 10 (5.6) 0 (0)	0.007*
**Place of delivery (previous birth)**	Home Health institution	18 (5.0) 342 (95.0)	38 (21.1) 142 (78.9)	0.000
**Mode of delivery (Previous birth)**	Vaginal Cesarean section	320 (88.8) 40 (11.1)	160 (88.9) 20 (11.1)	0.000
**Distance of health institutions**	< 15 min 15 – 30 min > 30 min	134 (37.2) 106 (29.4) 120 (33.3)	18 (10.0) 58 (32.2) 104 (57.8)	0.000
**Gestational age**	Term Preterm Post-term	308 (85.6) 24 (6.7) 28 (7.8)	162 (90.0) 12 (6.7) 6 (3.3)	0.133
**Contraception use**	Yes No	276 (76.7) 84 (23.3)	124 (68.9) 56 (31.1)	0.052
**Types of contraception used**	Injectable Implant IUCD OCPs Natural methods Tubal ligation	138 (50.0) 100 (36.2) 10 (3.6) 22 (8.0) 6 (2.2) 0 (0)	50 (40.3) 54 (43.5) 0 (0) 14 (11.3) 4 (3.2) 2 (1.6)	0.065*
**Planned pregnancy**	Yes No	336 (93.3) 24 (6.7)	152 (84.4) 28 (15.6)	0.001
**Postpartum counseling**	Yes No	308 (85.6) 52 (14.4)	136 (75.6) 44 (24.4)	0.040
**Alcohol use**	Yes No	38 (10.6) 322 (89.4)	14 (7.8) 166 (92.2)	0.302
**Newborn sex**	Male Female	190 (52.8) 170 (47.2)	100 (55.6) 80 (44.4)	0.542
**Birth weight**	Low birth weight Normal Macrosomic	38 (10.6) 294 (81.7) 28 (7.8)	20 (11.1) 148 (82.2) 12 (6.7)	0.887
**APGAR score**	Low Normal	22 (6.1) 338 (93.9)	18 (10.0) 162 (90.0)	0.104

*GA* Gestational age, *IUCD* Intrauterine contraception device, *IUFD* Intrauterine fetal death, *OCPs* Oral contraception pills, *SVD* Spontaneous vaginal delivery

^*^Fisher’s exact test

#### Adverse obstetric outcome

**Fig. 2:**
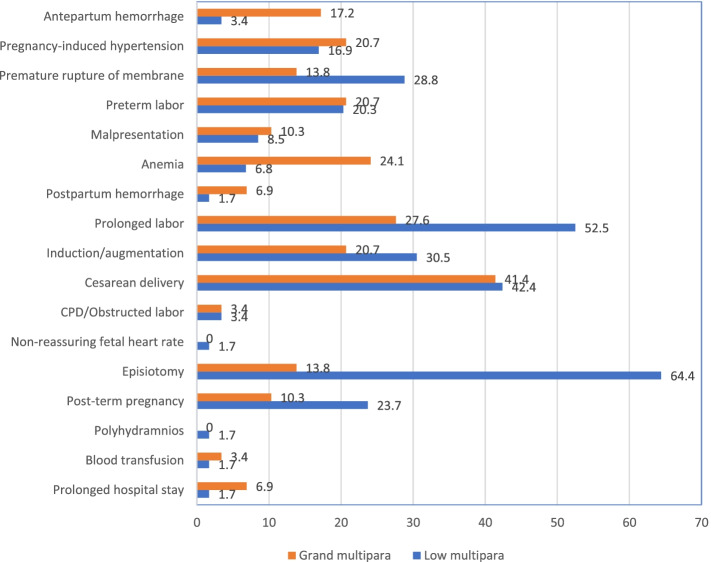
Adverse obstetric outcome in low multiparous and grand multiparous women in Public Hospitals of North Ethiopia, 2021

The prevalence of adverse obstetric outcomes was 32.6% (95% CI: 28.7–36.5). This was comparable in low multiparous and grand multiparous women (32.8% vs 32.2%). Antepartum hemorrhage, anemia, and postpartum hemorrhage were higher in grand multiparous women. Whereas, PROM, prolonged labor, induction/augmentation, episiotomy, and post-term pregnancy were higher in low multiparous women (Fig. 2).

### Determinants of adverse obstetric outcome

Variables with a p-value of ≤ 0.25 in the bivariable logistic regression analysis were selected for the multivariable logistic regression analysis model. When adjusted for the socio-demographic and obstetric factors, parity did not show a statistically significant difference in obstetric outcomes [AOR (CI) = 1.42 (0.74–2.73)]. However, income, alcohol consumption, gestational age at delivery, previous mode of delivery, and birth weight showed a statistically significant association (Table 4).

**Table 4:** Determinants of adverse obstetric outcome in Public Hospitals of North Ethiopia, 2021

Variables	Adverse obstetric outcome		COR (95% CI)	AOR (95% CI)
	**Yes**	**No**		
**Age**				
20 – 34 years	126 (71.6)	250 (68.70	1	1
< 20 years	8 (4.5)	4 (1.1)	3.97 (0.97–13.4)	3.44 (0.21–11.7)
≥ 35 years	42 (23.9)	110 (30.2)	0.76 (0.50–1.15)	0.67 (0.35–1.25)
**Religion**				
Christian	129 (73.3)	295 (81.0)	1	1
Muslim	47 (26.7)	69 (19.0)	1.56 (1.02–2.38)	2.01 (0.99–4.06)
**Mother’s education**				
No formal education	50 (28.4)	122 (33.5)	0.96 (0.61–1.49)	0.74 (0.29–1.87)
Primary	36 (20.5)	54 (14.8)	1.56 (0.93–2.61)	0.45 (0.17–1.21)
Secondary	30 (17.0)	48 (13.2)	1.46 (0.84–2.52)	0.82 (0.36–1.84)
Higher education	60 (34.1)	140 (38.5)	1	1
**Age at marriage**				
< 18 years	46 (26.1)	74 (20.3)	1.39 (0.91–2.12)	0.79 (0.38–1.64)
≥ 18 years	130 (73.9)	290 (79.9)	1	1
**Income (ETB)**				
Lower tertile	84 (47.7)	138 (37.9)	1.65 (1.06–2.58)	3.15 (1.30–7.63)*
Middle tertile	50 (28.4)	112 (30.8)	1.21 (0.75–1.97)	2.48 (1.22–5.06)*
Higher tertile	42 (23.9)	114 (31.3)	1	1
**GA at first ANC visit**				
≤ 16 weeks	101 (62.3)	177 (51.8)	1	1
> 16 weeks	61 (37.7)	165 (48.2)	0.65 (0.44–0.95)	0.37 (0.21–1.66)
**Previous obstetric complications**				
Yes	106 (60.2)	132 (36.3)	2.66 (1.84–3.85)	1.48 (0.88–2.49)
No	70 (39.8)	232 (63.7)	1	
**Mode of delivery (Previous birth)**				
Vaginal	128 (72.7)	352 (96.7)	1	1
Cesarean section	48 (27.3)	12 (3.3)	11.0 (5.66–21.4)	13.6 (6.18–30.1)*
**Planned pregnancy**				
Yes	146 (83.0)	342 (94.0)	1	1
No	30 (17.0)	22 (6.0)	3.19 (1.78–5.72)	1.25 (0.47–3.29)
**Alcohol consumption**				
Yes	24 (13.6)	28 (7.7)	1.89 (1.06–3.38)	3.15 (1.49–6.64)*
No	152 (86.4)	336 (92.3)	1	1
**GA at delivery**				
Term	128 (72.7)	342 (94.0)	1	1
Preterm	26 (14.8)	10 (2.7)	6.95 (3.26–14.8)	9.24 (2.28–27.3)*
Post-term	22 (12.5)	12 (3.3)	4.89 (2.36–10.2)	4.82 (1.89–12.3)*
**Newborn sex**				
Male	105 (59.7)	185 (50.8)	1.43 (0.99–2.06)	1.16 (0.71–1.92)
Female	71 (40.3)	179 (49.2)	1	1
**Birth weight**				
Normal	130 (73.9)	312 (85.7)	1	1
Low birth weight	34 (19.3)	24 (6.6)	3.40 (1.94–5.96)	3.46 (1.33–9.03)*
Macrosomic	12 (6.8)	28 (7.7)	1.03 (0.51–2.09)	0.53 (0.19–1.42)
**APGAR score**				
Low	24 (13.6)	16 (4.4)	3.43 (1.77–6.65)	1.51 (0.52–4.37)
Normal	152 (86.4)	348 (95.6)	1	1
**Parity**				
Low multiparous	118 (67.0)	242 (66.5)	1	1
Grand multiparous	58 (33.0)	122 (33.5)	0.97 (0.67–1.43)	1.42 (0.74–2.73)

*SVD* Spontaneous vaginal delivery, *GA* Gestational age

^*^Statistically significant at p-value < 0.05

Mothers in the low-income tertile were three times more likely to develop obstetric complications compared to those in higher-income tertiles (AOR (CI) = 3.15 (1.30–7.63). The odds of adverse obstetric outcomes were higher among cesarean deliveries than the vaginal (AOR (CI) = 13.6 (6.18–30.1). Alcohol consumption increased the risk of adverse obstetric outcomes by threefold (AOR (CI) = 3.15 (1.49–6.64). Adverse obstetric outcomes were nine times more common in women with preterm deliveries (AOR (CI) = 9.24 (2.28–27.3). Further, mothers with low-birth-weight neonates were three times more likely to have adverse obstetric outcomes compared to normal birth weight (AOR (CI) = 3.46 (1.33–9.03) (Table 4).

## Discussion

This study compared the adverse obstetric outcomes in low multiparous and grand multiparous women. APH, anemia, and PPH were higher in grand multiparous women. Whereas, PROM, prolonged labor, induction/augmentation, episiotomy, and post-term pregnancy were higher in low multiparous women. Income, alcohol consumption, gestational age at delivery, previous mode of delivery, and birth weight were significant predictors of adverse obstetric outcomes. However, parity showed an insignificant difference in obstetric outcomes.

The current study found that parity was not significantly associated with composite adverse obstetric outcomes. However, APH, anemia, and PPH were higher in grand multiparous women. Whereas, PROM, prolonged labor, induction/augmentation, episiotomy, and post-term pregnancy were higher in low multiparous women. Similarly, a systematic review and meta-analysis finding showed that grand multiparity was not associated with an increased risk of pregnancy outcomes [16]. In Saudi, there is an insignificant increase in the maternal and neonatal risks in grand multiparas compared to the low multiparas. The study further concluded that grand multiparity could not be discouraged given that the women are provided with good perinatal care [7]. A comparative prospective cohort study in Uganda also reported that there was no difference in fetal outcome between grand multiparous and low multiparous women [17]. Further, grand multiparity was found to be an insignificant factor for adverse obstetric outcomes in South Ethiopia [6].

On the contrary, other studies found a statistically significant association between grand multiparity and adverse obstetric outcomes [9, 18, 19]. Grand multiparity was considered a risk pregnancy and increased the risk of obstetric complications in Tanzania [15]. These might be because of the variation in study design, setting, socio-economic status, and lack of account for possible confounders, i.e., interpregnancy interval, chronic disease, nutritional and psychosocial status. Besides, significant outcomes in the previous studies might be related to low health service utilization of grand multiparous women. Further, the differences in antenatal care access and quality may explain this disparity.

According to the current study, mothers in the low-income tertile were three times more likely to develop obstetric complications compared to those in higher-income tertiles. In Korea, the risk of obstetric complications, i.e., cesarean delivery, pre-eclampsia, gestational diabetes, obstetric hemorrhage, and preterm delivery were significantly higher in women with low-income levels [20]. Mothers with low-income levels also had higher risks of death [21]. This could be because women with low socioeconomic status tend to have low educational levels, inadequate prenatal visits, and poor medical service utilization. In addition, prolonged working hours/occupational fatigue and physical exertions likely affect obstetric outcomes.

It was found that the mode of delivery had a positive association with adverse obstetric outcomes. The odds of adverse obstetric outcomes were significantly higher among cesarean deliveries. A prospective cohort in Nepal found that the presence of severe obstetric complications significantly increased the likelihood of cesarean delivery [22]. Cesarean delivery appeared to meet the obstetric need to save the life of the mother and/or fetus and was performed following medical indications, particularly after the onset of labor. The most common obstetric indications of cesarean delivery were malpresentation, prolonged labor, non-reassuring fetal heart rate pattern, and obstructed labor.

The current study identified alcohol consumption as a risk factor for adverse obstetric outcomes. Alcohol consumption increased the risk of adverse obstetric outcomes by threefold. This is comparable with a prospective cohort study in Japan that found alcohol consumption was associated with an increased risk of preterm birth [23]. In addition, women who drink alcohol had significantly higher odds of pregnancy-induced hypertension (PIH) [24]. The mechanism of this link might be due to alcohol induces endothelial dysfunction and insufficient spiral artery remodeling resulting in severe intravascular coagulation, decreased placental perfusion, placental dysfunction, and an imbalance of endogenous angiogenic factors, such as soluble fms-like tyrosine kinase 1 (sFlt-1) and placental growth factor (PlGF). Alcohol could also increase the secretion of prostaglandins that increase cyclic 3ʹ,5ʹ-adenosine monophosphate activity and yield decreased cell division and increased uterine contractions [23, 24].

Gestational age at delivery was found to be an associated risk factor for obstetric complications. Women with preterm deliveries were nine times at higher risk of adverse obstetric outcomes. This finding was supported by a study done in Western Ethiopia, where mothers who developed anemia during pregnancy, PROM, and PIH were more likely to experience preterm birth [25]. Anemia may induce maternal and fetal stress and increase the risk of maternal infection stimulating the production of corticotropin-releasing hormone (CRH). Elevated CRH is a major risk factor for preterm labor, premature rupture of the membranes, and pregnancy-induced hypertension and eclampsia [26]. As amniotic fluid contains prostaglandin, PROM elevates fetal plasma interleukin-6 and induces uterine contraction. PIH may cause vascular damage to the placenta causing antenatal bleeding and preterm birth.

Moreover, mothers with low-birth-weight neonates were three times more likely to have adverse obstetric outcomes compared to normal birth weight neonates. Similarly, a cross-sectional study in Wolaita Sodo found that pregnancy-induced hypertension and anemia during pregnancy have independent effects in causing low birth weight [27]. Secondary data analysis in Zimbabwe also stated that the risk of low birth weight was significantly higher among women with PROM, eclampsia, anemia, APH, and preterm labor [28]. Low birth weight indicates the presence of some kind of obstetric complication that adversely affects the growth of the fetus. For example, hypertension in pregnancy may cause abruption placenta, which might result in reduced nutrient and oxygen supply to the growing fetus and may end up in low birth weight, growth restriction, or stillbirth [29]. This might be also due to poor socioeconomic status, inadequate maternal nutrition, and weight gain during pregnancy. This finding may also call attention to early identification and treatment of pregnancy complications and launch the 2016 WHO global recommendations for routine ANC visits.

### Limitation

This finding study should be interpreted with the following drawbacks. Due to the insufficient count of cases, it was not possible to examine each specific adverse obstetric outcome separately with parity. There may be also a recall bias on previous obstetric profiles. Since it is a snapshot, it shares the limitation of a cross-sectional study that may not indicate a causal relationship. Finally, as the study was done in a hospital setting, the obstetric outcome of women who gave birth at home was not assessed.

## Conclusion and recommendation

The adverse obstetric outcome was significantly associated with income, alcohol consumption, preterm delivery, cesarean delivery, and birth weight. Parity had no significant association with obstetric outcomes. Socio-economic development, avoiding alcohol consumption, early identification and treatment of complications, and adequate nutrition and weight gain during pregnancy are needed regardless of parity. Attention should be paid to both groups of women for their different obstetric complications. In addition, longitudinal studies are recommended to investigate the effect of parity on adverse obstetric outcomes.

## Data Availability

The data sets are available from the corresponding author and can be shared upon reasonable request.

## Abbreviations

ANC: Antenatal care
APH: Antepartum Hemorrhage
DBU: Debre Berhan University
GM: Grand Multipara
IUFD: Intrauterine Fetal Death
LM: Low Multipara
PPH: Postpartum Hemorrhage
PROM: Prelabor rupture of membrane
